# Detection of low frequency artemisinin resistance mutations, C469Y, P553L and A675V, and fixed antifolate resistance mutations in asymptomatic primary school children in Kenya

**DOI:** 10.1186/s12879-025-10462-z

**Published:** 2025-01-16

**Authors:** Victor Osoti, Kevin Wamae, Leonard Ndwiga, Paul M. Gichuki, Collins Okoyo, Stella Kepha, Kibor Keitany, Regina Kandie, Stephen Aricha, Rosebella Kiplagat, Charles Mwandawiro, Philip Bejon, Robert W. Snow, Lynette Isabella Ochola-Oyier

**Affiliations:** 1 Centre for Geographic Medicine Research (Coast), Kenya Medical Research Institute-Wellcome Trust Research Programme, Kilifi, Kenya; 2 Eastern and Southern Africa Centre of International Parasite Control, Kenya Medical Research Institute, Nairobi, Kenya; 3 Centre for Tropical Medicine and Global Health, Nuffield Department of Medicine, University of Oxford, Oxford, UK; 4 Division of National Malaria Programme, Ministry of Health, Nairobi, Kenya

**Keywords:** *Plasmodium Falciparum*, Genomic surveillance, Amplicon deep sequencing, Artemisinin resistance

## Abstract

**Background:**

To understand the emergence and spread of drug-resistant parasites in malaria-endemic areas, accurate assessment and monitoring of antimalarial drug resistance markers is critical. Recent advances in next-generation sequencing (NGS) technologies have enabled the tracking of drug-resistant malaria parasites.

**Methods:**

In this study, we used Targeted Amplicon Deep Sequencing (TADS) to characterise the genetic diversity of the *Pfk13*, *Pfdhfr*, *Pfdhps*, and *Pfmdr1* genes among primary school-going children in 15 counties in Kenya (Bungoma, Busia, Homa Bay, Migori, Kakamega, Kilifi, Kirinyaga, Kisii, Kisumu, Kwale, Siaya, Tana River, Turkana, Vihiga and West Pokot). A total of 920 dried blood spot (DBS) samples collected from 121 selected primary schools within the country were used to extract genomic DNA. A nested polymerase chain reaction (PCR) was used to generate amplicons that were sequenced to determine the prevalence of known and novel polymorphisms.

**Results:**

*Pfk13* mutations associated with artemisinin resistance were present as mixed genotype infections for the C469Y mutation in 23 samples (4%), the A675V mutation in 2 samples (1.7%), and the P553L mutation in 7 samples (1.2%). The A578S mutation, was also identified in mixed infections, appearing in 15.2% of the 87 samples analysed. The *Pfdhfr* 51I and 108 N pyrimethamine-resistance mutations were at fixation (100% frequency), and the *Pfmdr1* Y184F mutation, which is linked to reduced susceptibility to several antimalarial drugs, especially those used in combination therapies for malaria treatment, was detected in 97.5% of the samples as mixed-genotype infections.

**Conclusion:**

The genomic surveillance of asymptomatic school children in Kenya provides an early warning signal of at least 1 of the 3 validated artemisinin resistance mutations circulating in all regions in Western Kenya sampled except Homa Bay and Kisii Counties. These signals in asymptomatic and mixed infections would have been missed without deep sequencing.

**Supplementary Information:**

The online version contains supplementary material available at 10.1186/s12879-025-10462-z.

## Background

Resistance to antimalarial drugs challenges our ability to manage acute malaria and eliminate the burden that malaria places on individuals and societies. Molecular marker studies can be used to track the prevalence of key molecular mutations that are known to confer resistance (Diakité et al. 2019), which in turn enables timely action to prevent its spread and limit public health impacts.

A high rate of ACT failure with dihydroartemisinin-piperaquine (DHA-PPQ) has been documented in the Greater Mekong subregion (GMS), and mutations associated with resistance to the ACT partner drug piperaquine have been documented (WHO 2019).

Multiple single nucleotide polymorphisms (SNPs) in the Kelch13 (*k13*) gene, such as C580Y, F446I, C469Y, Y493H, R539T, I543T, P553L, R561H, P574L, M579I, A675V and R622I, have been strongly linked to clinical artemisinin resistance (Bayih et al. 2016; Conrad and Rosenthal 2019; Uwimana et al. 2020). Further mutations in the *Pfk13* propeller domain have been linked to artemisinin partial resistance in Africa. Recent work has revealed clonal expansion of the R622I and R561H mutations, which have been linked to delayed parasite clearance in ACT-treated patients in Ethiopia and Rwanda, respectively (Alemayehu et al. 2021; Uwimana et al. 2020). C469Y and A675V, ART-R candidate markers, were also found in more than 15% of Ugandan samples from 2018 to 2019 (Balikagala et al. 2021). Parasites with the C469Y mutation have been confirmed to show reduced in vitro susceptibility to artemisinin compared to wild-type controls. The haplotypic network analysis revealed that the flanking regions of the C469Y mutation share the same African genetic background, suggesting a single, indigenous origin of the mutation (Awor et al. 2024). In addition, these mutations are now more than 20% in the population, initially in Northern Uganda and recently in the East and West of the country with the inclusion of additional mutations, R561H and C469F (Conrad et al. 2023). Other validated *Pfk13* mutations have been reported in Africa, although at low frequencies, these include F446I, M476I, P553L, P574L, C580Y, and C469F (Owoloye et al. 2021). The World Health Organization (WHO) has identified eight mutations as potential candidate markers for drug resistance: P441L, G449A, C469F, A481V, R515K, P527H, G538V, and V568G (World Health Organization 2022).

While mutations in the *Pfk13* gene are widely recognized as key determinants of artemisinin resistance in *Plasmodium falciparum*, emerging evidence highlights additional genetic and biochemical mechanisms that contribute to this resistance. Mutations in the *Pfcoronin* gene have been associated with delayed parasite clearance and enhanced survival following exposure to dihydroartemisinin (DHA), acting independently of *PfK13* by influencing parasite fitness and drug response (Tumwebaze et al. 2022). Variants in the *ap2µ* and *ubp1* genes have also been implicated in artemisinin resistance through their effects on hemoglobin uptake and metabolism, where altered hemoglobin processing may reduce artemisinin activation and decrease its efficacy (Zheng et al. 2024). In addition to these genetic mutations, resistant *P. falciparum* strains exhibit enhanced adaptive responses to oxidative stress, enabling parasites to mitigate protein damage caused by artemisinin exposure and promoting their survival under drug pressure (Hanboonkunupakarn et al. 2022). Moreover, the susceptibility of *P. falciparum* to artemisinin is highly stage-specific, with ring-stage parasites being particularly vulnerable compared to mature stages, which are less susceptible and may contribute to treatment failures (Hanboonkunupakarn et al. 2022). These alternative mechanisms, acting in parallel or in synergy with *Pfk13* mutations, underscore the multifactorial nature of artemisinin resistance in *P. falciparum* and highlight the importance of understanding these pathways to develop comprehensive strategies for monitoring and combating resistance in malaria-endemic regions.

Pyrimethamine resistance has been linked to several mutations in the *P. falciparum* dihydrofolate-reductase *(Pfdhfr*) gene, including the *Pfdhfr* 108 N, and additional synergistic mutations at codons 51I, 59R, 164 L (Zakeri et al. 2007) that increase resistance to pyrimethamine (Gregson and Plowe 2005; Peterson et al. 1988). Further combinations of substitutions at codons 436 A, 437G, 540E, and 581G of the *P. falciparum* dihydropteroate synthetase (*Pfdhps*), similarly synergistically increase resistance to sulfadoxine (Wang et al. 1997). In Kenya, there is a long standing high prevalence of *Pfdhfr* and *Pfdhps* mutations (Iriemenam et al. 2012).

The *Plasmodium falciparum* multidrug resistance gene 1 (*Pfmdr1*) influences susceptibility to various antimalarial drugs, including chloroquine (CQ), lumefantrine (LMF), amodiaquine (AQ), and artemisinin (AS) (Duraisingh and Cowman 2005; Veiga et al. 2016). Key mutations, such as N86Y, Y184F, S1034C, N1042D, and D1246Y, have been linked to resistance (Wicht et al. 2020). The N86Y mutation, prevalent in Asia and Africa, increases resistance to CQ and AQ, while enhancing susceptibility to LMF, mefloquine (MFQ), and dihydroartemisinin (DHA) (Huang et al. 2016; Veiga et al. 2016). A decline in the 86Y mutation has been observed following the adoption of artemisinin-based combination therapies (ACTs) (Lobo et al. 2014; Maiga et al. 2021) and the cessation of chloroquine use. There is an urgent and growing concern about increasing artemisinin resistance mutations in the East and Horn of Africa region. This study presents malaria molecular surveillance data from infected, asymptomatic school children across Kenya.

## Methods

### Study design and setting

**Fig. 1:**
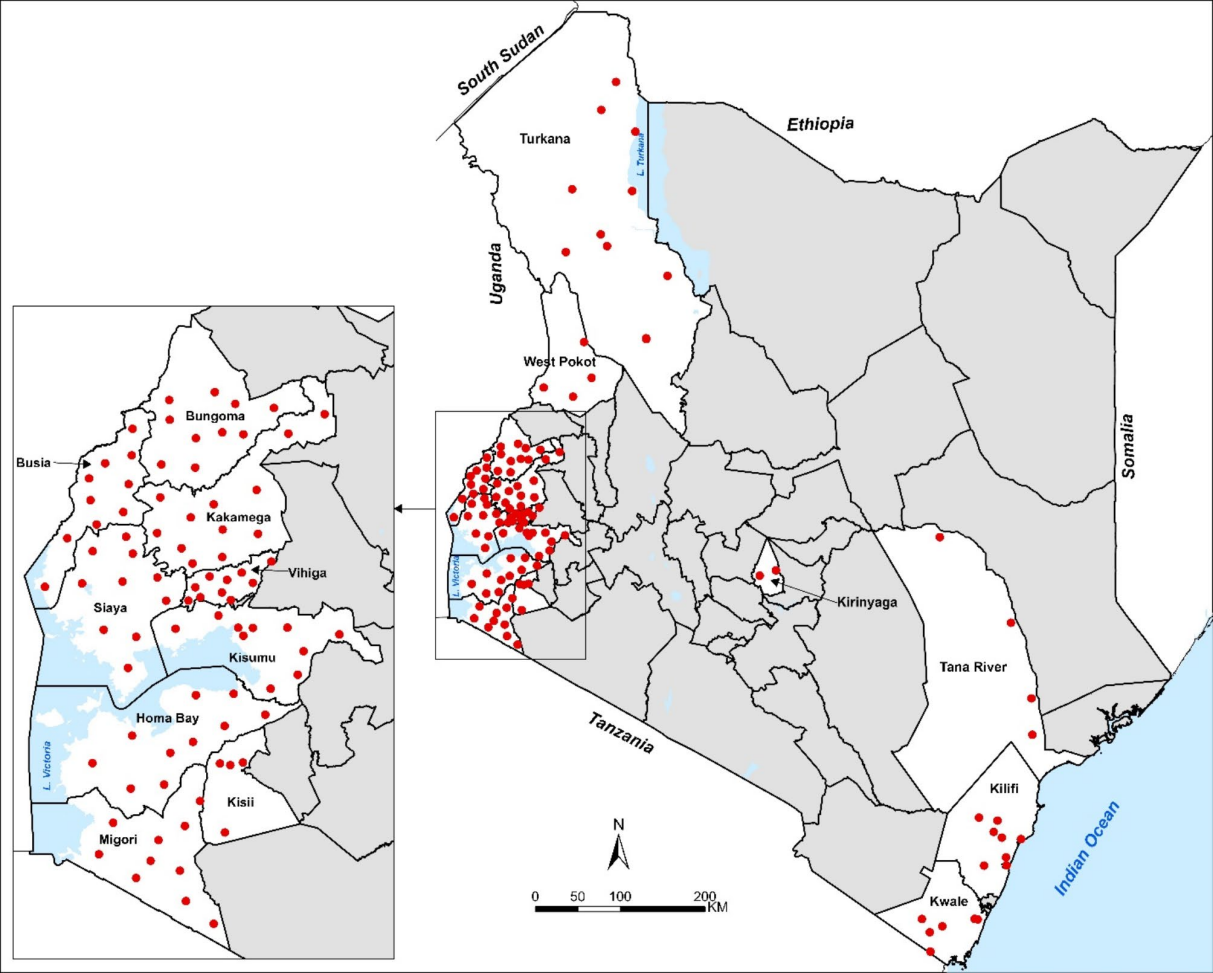
A map of Kenya showing the counties and schools sampled. The red dots indicate the schools sampled in this study. All malaria DBS samples were collected within a school deworming project across all 15 counties

Kenya is characterised by a diverse malaria ecology, ranging from semi-arid, sporadic transmission, epidemic prone highland malaria, coastal intermediary intensity transmission and high transmission areas surrounding Lake Victoria (Alegana et al. 2021). This study leveraged previous work that surveyed the prevalence and distribution of malaria infection among school children in Kenya from March to November 2022 across 45 of the 47 counties in Kenya (Alegana et al. 2021). For the current antimalarial drug-resistance surveillance study, we employed an opportunistic sampling strategy alongside this previous work whereby we included only 15 counties across malaria-endemic regions: Bungoma, Busia, Homa Bay, Kakamega, Kisumu, Migori, Siaya, and Vihiga, (lakeside), Kisii (highland), Kilifi and Kwale (Coastal), Kirinyaga (low risk), Turkana, Tana River and West Pokot (semi-arid) (Fig. 1). 121 schools were included in the current study as shown in Table 1. Dried blood spots (DBS) were collected from all participants from the 15 counties, but only the rapid diagnostic test (RDT) positive samples were processed for sequencing.

School-based surveys were undertaken using sampling methods described elsewhere (Osoti et al. 2022). In brief, schools were randomly selected from each county in proportion to the total number of schools within that county. On the survey day, 100 children aged 5–14 years were randomly selected from the school register. An initial meeting was held with the head teacher, school committee, and parents to explain the study, and before enrolment, the student’s parents or guardians provided informed consent while each child assented. Instead of written opt-in consent, parental consent was based on passive, opt-out consent (Ellickson and Hawes 1989). A finger prick was taken to obtain blood for malaria rapid diagnostic test (mRDT) (CareStart™), and a Dried Blood Spot Sample (DBS) was also collected on a Whatman CF12 filter paper (Cat No. 10535097, Cytiva, USA) for molecular diagnosis. Clinical assessments were conducted, and a structured questionnaire was administered for each child to gather information about their recent health history. The questionnaire included questions about hospital admissions, school absences due to illness, episodes of fever, and the use of antimalarial drugs within the past month. After allowing the DBS samples to air dry for at least 1 h, they were individually packed in zip-lock bags with a desiccant and shipped to the KEMRI-Wellcome Trust Research Programme laboratories. Children who tested mRDT positive for malaria were treated following the national malaria treatment guidelines with artemether-lumefantrine (AL) as a co-formulated tablet of 20 mg of artemether and 120 mg of lumefantrine. The study was approved by the KEMRI and National Ethics Review Committee (number KEMRI/SERU/ESACIPAC/11/3822). Additional approval was provided by the county health and education authorities.

### DNA extraction, and amplicon deep sequencing (AmpSeq)

To extract parasite DNA from the RDT-positive DBS, a previously published protocol (Osoti et al. 2022) was used. Briefly, 4 punches 6 mm each were punched from two locations (at the center and periphery) of the DBS and placed into a 1.5 ml Eppendorf tube using sterile tweezers. DNA extraction was done using the Chelex saponin method (Baidjoe et al. 2013). Parasite DNA was amplified using 18 S rRNA *Plasmodium falciparum* qPCR assay (Hermsen et al. 2001). Samples from Kakamega, Busia, Homa Bay, Kisumu, Migori, and Siaya with a median cycling threshold (Ct) above 31.5 to select samples with a high parasite density to increase the success of amplification and to reduce the numbers of samples sequenced from this location that generated a large amplicon sample size. In contrast, for samples from Bungoma, Kwale, Kisii, Vihiga, Turkana, and West Pokot, a minimum Ct threshold of 38.5 was set, since the amplicon sample size was low, and this Ct would still allow for the detection of parasites even with low parasite densities.

Using a previously published nested PCR approach (Osoti et al. 2022) amplicons were generated for the following genes, using molecular identifiers (MID) labelled primers (Osoti et al. 2022) for *P. falciparum* dihydrofolate reductase (*Pfdhfr*: PF3D7_0417200), P. *falciparum* dihydropteroate synthase (*Pfdhps*: PF3D7_0810800), *P. falciparum* kelch13 (*Pfk13*: PF3D7_1343700) and *P. falciparum* multidrug resistance 1 (*Pfmdr1*: PF3D7_0523000). The *dhfr*,* dhps* and *mdr1* genes serve as controls for the assays since the mutation loci and frequencies are known from previous data (Osoti et al. 2022). Slight modifications were made to the initial protocol, generating PCR amplicons, and sequencing them in singlets rather than duplicates. The genotyping focused on specific mutations within the Kelch propeller domain of the *k13* gene, which are essential for monitoring artemisinin resistance. One set of primers covered a fragment spanning positions 2156 bp (codon 719) to 2645 bp (codon 882), while the other set covered a fragment spanning positions 2509 bp (codon 837) to 3020 bp (codon 1007). The amplification used external PCR primers *Pfk13*ext675F (5’-GAAGCCTTGTTGAAAGAAGC-3’) and *Pfk13*ext675R (5’-CGGAGTGACCAAATCTGG-3’), and internal PCR primers *Pfk13*int675F (5’-GGGGGATATGATGGCTCTTCT-3’) and *Pfk13*int675R (5’-ACTAATAAAGATGGGCCAAGC-3’).

The PCR products were individually purified using the AMPure XP beads (Beckman Coulter, Inc.) as per the manufacturer’s instructions. Thereafter, the purified DNA was quantified using a Qubit double-strand DNA (dsDNA) high-sensitivity (HS) assay kit, according to the manufacturer’s instructions. Library preparation was done using the KAPA kit, while a size selection clean-up was done using 0.8X AMPure XP beads. The adapter-ligated libraries were amplified using Illumina primers and cleaned with 0.8X AMPure. The libraries were quantified using a Qubit dsDNA HS kit and sizes were verified by the DNA 1000 assay kit using the 2100 Bioanalyzer (Agilent). The libraries were mixed in equimolar concentrations, denatured, spiked with 8% PhiX DNA, and finally AmpSeq was done using a MiSeq reagent kit v3 (Illumina). The entire procedure has been reported by Osoti et al. (2022).

### Sequence data analysis

SeekDeep v3.0.1 initially demultiplexed the sequences based on the MIDs. The paired consensus reads were trimmed and clustered to estimate the frequency of DNA clusters (referred to as microhaplotypes from here henceforth). Microhaplotypes were discarded if their relative frequency was < 5%, based on previous published work (Osoti et al. 2022) that set this as the limit of detection to confidently call microhaplotypes. Chimeric reads were considered PCR artefacts and discarded. The SNP and microhaplotype frequencies in the population were calculated as the number of samples containing the SNP or microhaplotype over the total number of samples genotyped to determine the number of individuals harbouring a SNP or haplotype. All statistical analysis were performed in R v4.0.3 (R Core Team 2022).

## Results

A total of 10,873 students from 121 primary schools from the 15 counties sampled in Kenya (Table 1) were screened and 1742 were malaria positive by RDT. The dried blood spots (DBS) from RDT-positive children were screened by 18 S rRNA *Plasmodium falciparum* qPCR assay to identify 920 samples with detectable levels of DNA to take forward for AmpSeq (Table 1). We obtained sequences from 660 samples for *Pfdhfr*, 756 samples for *Pfdhps*, 571 samples for *Pfk13* at codon 469, 119 samples for *Pfk13* at codon 675, and 518 samples of *Pfmdr1* (Table 2). Notably, due to the variation in the sequencing runs, the sequencing success for *Pfk13* codon 675 was much lower, from fewer samples.

**Table 1:** The samples collected between March 2022 and November 2022 for the *P. falciparum* drug resistance marker genotyping

Study Site	Time point	Schools sampled	Samples collected	mRDT No. [%]	DNA extraction	18s Pf pos, No. [%]	No. of PCR amplicons
Bungoma	Mar/Apr	10	987	74 [8]	74	54[73]	54
Busia	Mar/Apr	10	989	322 [33]	322	276[85]	181
Homabay	Mar/Apr	10	979	174 [18]	174	86[49]	55
Kakamega	Mar/Apr	12	1195	145 [12]	145	106[73]	69
Siaya	Mar/Apr	10	993	337 [34]	337	277[82]	201
Vihiga	Mar/Apr	10	996	68 [7]	68	51[75]	51
Kisumu	Mar/Apr	10	966	161 [17]	161	126[78]	92
Migori	Mar/Apr	10	981	275 [28]	275	206[75]	137
Kisii	May/Jun	4	182	12 [7]	12	12[100]	10
Kwale	May/Jun	6	373	18 [5]	18	8[44]	8
Kilifi	May/Jun	8	541	1 [0.2]	1	1[100]	0
Kirinyaga	May/Jun	2	135	0	0	0	0
Tana River	May/Jun	4	246	0	0	0	0
Turkana	Oct/Nov	11	1024	92 [9]	92	42[46]	42
West Pokot	Oct/Nov	4	286	32 [11]	32	20[63]	20
**Total**		**121**	**10,873**	**1711**	**1711**	**1260**	**920**

### Identification and distribution of artemisinin resistance mutations

We successfully amplified and sequenced 690 samples, covering all the validated *Pfk13* artemisinin resistance mutations. Mutations in four different positions were detected, all only observed in mixed genotype infections. The most prevalent mutation (A578S) was detected in 87 samples (15.2%), C469Y in 23 samples (4%), A675V in 2 samples (1.7%) and the P553L mutation was detected in 7 samples (1.2%) (Table 2). The C469Y, P553L and A675V mutations are validated artemisinin resistance markers.

**Table 2:** Frequency of mutations for each drug-resistant gene in the population

			Frequency (%)		
Gene	Mutation	No. successfully sequenced	Wild type	Mutant	Mixed
dhfr	N51I	660	0	99.2	0.8
	C59R		0.6	80.8	18.6
	S108N		0	99.7	0.3
	I164L		76.1	0	23.9
dhps	S436A/H	756	57.8	1.2	40.5
	A437G		0.8	93.8	5.4
	P477S		99.2	0	0.8
	K540E		0.1	95.2	4.6
	A581G		96.2	0	3.8
K13	P441A	571	98.9	0	1.1
	G453C		98.8	0	1.2
	C469Y		96	0	4
	N489K		97.7	0	2.3
	N499S		99.1	0	0.9
	A504V		98.8	0	1.2
	V517I		98.2	0	1.8
	S522C		96.7	0	3.3
	N537S		99.6	0	0.4
	P553S		97.5	0	1.2
	P553L		97.5	0	1.2
	Y558C		97.4	0	2.6
	A569T		98.1	0	1.9
	A578S		84.8	0	15.2
	P667S	119	93.3	0	6.7
	A675V		98.3	0	1.7
	S679T		97.5	0	2.5
	E691D		12.6	0	87.4
mdr1	N86Y	518	100	0	0
	Y184F		1.5	1.2	97.5
	T199S		72.2	0	27.8

The rows highlighted in grey are the WHO k13 validated mutations

**Fig. 2:**
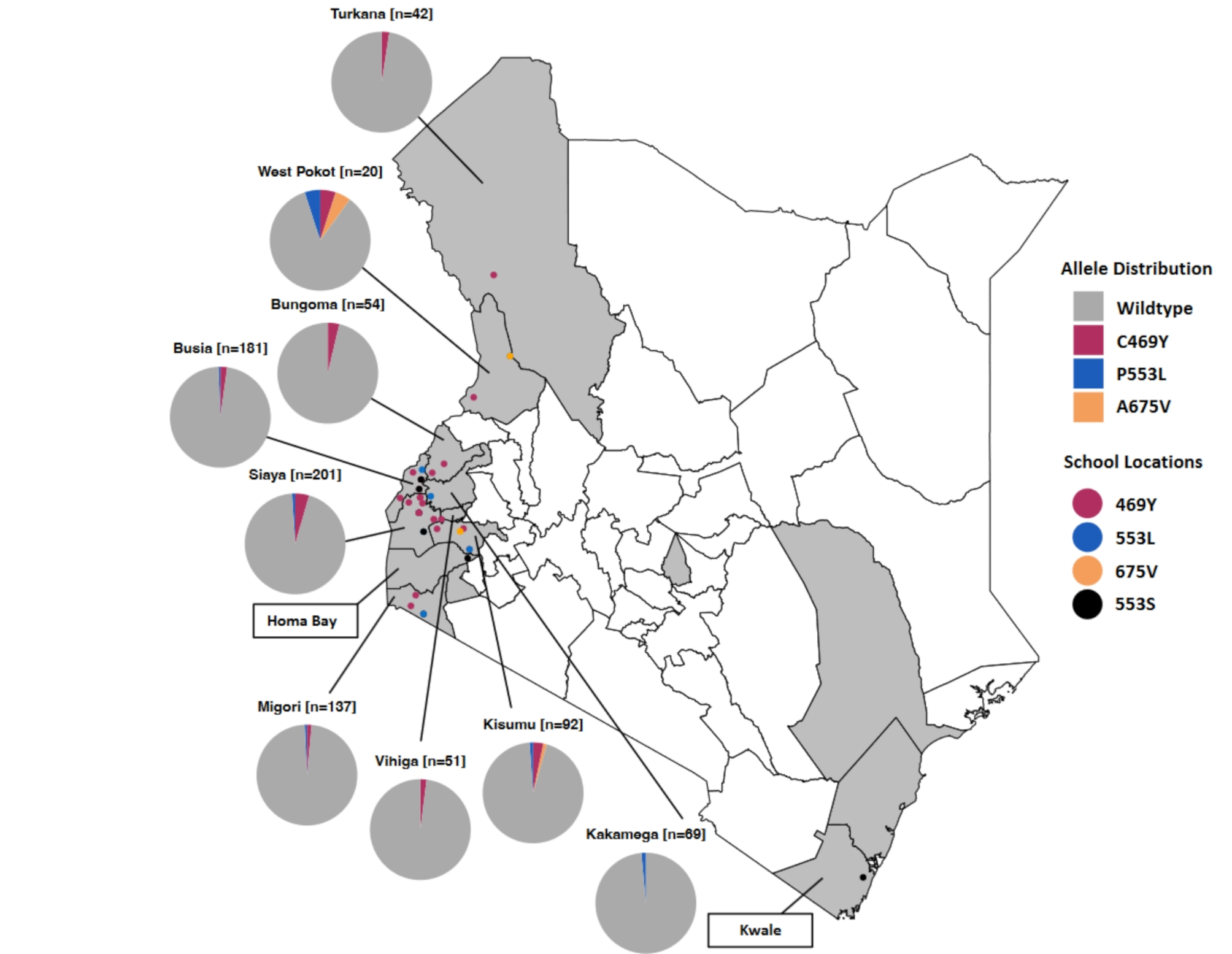
A map of Kenya showing the distribution of *k13* artemisinin drug resistance mutations across the country. The circles indicate the locations of schools where samples were collected, with each circle colour showing the mutation identified at that site. The circle colours match the colours in the pie chart that corresponds to the proportion of the specific k13 mutations: blue represents the P553L, orange denotes the A675V, red indicates the C469Y, black represents the P553S mutation, and grey represents wild-type.

The occurrence of the C469Y mutation was highest in Siaya County with 9 samples, followed by Busia with 4, Kisumu 3, and Bungoma and Migori with 2 samples each, West Pokot, Turkana, and Vihiga counties each had 1 sample with the C469Y mutation (Fig. 2). The 675 V mutation was detected in a sample each from Kisumu and West Pokot. Two samples with the P553L mutation were identified in Siaya County, while Busia, Kakamega, Kisumu, Migori, and West Pokot each had one sample with this mutation. An additional alternate mutation at codon 553, P553S, though not known to be associated with resistance, was observed in 1 sample each in Busia, Homa Bay, Kwale and Siaya counties (Fig. 2). No mutations were found at any of the other confirmed artemisinin resistance-conferring loci, but 14 rare mutations were observed as described in Table 2.

### Other genetic markers of antimalarial resistance

Codons 51, 59, 108, and 164 of the *Pfdhfr* gene were genotyped. The mutations in codons 51I and 108 N were fixed in the population (no wildtype only infections were identified). The 164 L mutation was detected as mixed genotype containing wild-type sequences (23.9%) (Table 2). The quadruple mutant (51I, 59R, 108 N, 164 L) was present in < 5% of the parasite population (Table 3, while the common *Pfdhfr* triple mutant (51I, 59R, 108 N) was found in 92% of the study population. Five *Pfdhps* mutations were identified, 437G (93.8% mutant and 5.4% as mixed infection) (Table 2) and 540E (95.2% mutant and 4.6% as mixed infection) were fixed with no wildtype only infections. Codon 436 A/H (40.5% as mixed infection) had similar frequencies of wildtype only and mixed infections, and emerging mutant only infections. The other 2 mutations (codons 477 and 581) were at a low frequency (Table 2).

Three SNP loci in *Pfmdr1* were genotyped, resulting in two microhaplotypes, since codon 86 was 100% only wild type (Table 3). Most parasite genotypes were a mix of wild-type (i.e., NYT) and mutant (NFT/NYS/NFS) microhaplotypes. The *Pfdhps* SGEA double mutant microhaplotype was the most prevalent at 88.2%, and the *Pfdhps* SGEG triple mutant haplotype was rare at 0.4% (Table 3). An adjusted microhaplotype frequency weighted by the within-host frequencies has been included in Supplementary Table S1, demonstrating a similarity in the predominant alleles in the population of the *dhfr*-IRNI, *dhps*- SGEA and *mdr1*-NYT/NFT microhaplotypes as the assessment of the frequencies per individual.

**Table 3:** Frequency of resistance haplotypes for each gene

Gene (Codons)	Haplotype	*n*	Frequency %
***dhfr*** **(51**,**59**,**108**,**164**)	IRNI	607	92
	ICNI	25	3.8
(*n* = 660)	IRNL	15	2.3
	ICNL	11	1.7
***dhps*** **(436**,** 437**,**540**,**581)**	HGEA	71	9.4
	SGEA	667	88.2
	SAKA	15	2
(*n* = 756)	SGEG	3	0.4
***mdr1*** **(86**,**184**,**199)**	NYT	571	45.8
	NFT	531	42.6
(*n* = 518)	NYS	137	11
	NFS	8	0.6

The frequency percentage of each haplotype is determined by the number of occurrences of a particular microhaplotype divided by the total number of microhaplotypes, denoted as ‘n’

## Discussion

Three validated *Pfk13* mutations, C469Y, P553L and A675V, were detected in the Western part of Kenya. These mutations have been validated and are linked with artemisinin resistance ( Uwimana et al. 2020; Balikagala et al. 2021; Owoloye et al. 2021). The A578S was the most prevalent SNP observed in our study and has been observed in other sub-Saharan African countries like Ghana, Kenya, Gabon, DRC, Uganda, Cameroon, and Mali (Kamau et al. 2015; Li et al. 2019; WWARN K13 Genotype-Phenotype Study Group 2019). However, the A578S mutation is not associated with artemisinin resistance (Ménard et al. 2016). The A675V mutation was recently identified in Kakamega county in 3 samples from 2021, also from asymptomatic school children (Jeang et al. 2024) and C469Y in Busia, from 3 samples but from symptomatic individuals (Makau et al. 2024). Previous studies (Balikagala et al. 2021) have shown an association between prolonged parasite clearance half-lives after artemisinin monotherapy in infections containing the C469Y and A675V mutations. The continued emergence of artemisinin resistance in Africa will likely slow down the gains in malaria treatment and control.

The rare S522C mutation identified in the current study (3.3%) as mixed infections has previously been reported in Kenya (Schmedes et al. 2021), in two samples collected during a Therapeutic Efficacy Study (TES) between 2016 and 2017. The World Health Organization (WHO 2018) has also reported this mutation to be associated with delayed parasite clearance. However, due to limited data, it was unclear if criteria for statistical significance were fully met.

A large majority of the emerging mutations in the asymptomatic infections were observed in mixed infections. This is common in areas of high malaria transmission where the complexity of infection is high (Kimenyi et al. 2022; Ogwang et al. 2024; Wamae et al. 2022). Potential contamination was ruled out based on the inclusion of genes with known resistance mutations such as *dhfr*,* dhps* and *mdr1* whose mutation frequencies reflect what is known due to the current selection pressure. For instance, *mdr1* codon 86 was 100% wildtype (N86) with no mutant or mixed infections since the cessation of chloroquine use that selected for the resistance genotype. Codon 108 in *dhfr* is 0% wildtype and 99.7% solely mutant, with only 0.3% mixed infections, reflecting the low survival of wild-type only mutations in the Kenya population where SP is still in use. The emerging mutations or low frequency variants, such as those observed in *k13* or *dhfr* 164 L, were present in mixed infections potentially due to developing selection pressure that eventually generates mutant only infections.

Three *dhfr* mutations N51I, C59R, S108N and two *dhps* mutations A437G, K540E (i.e., the quintuple mutation associated with resistance to the antifolates pyrimethamine and sulfadoxine) were at very high prevalence and the wildtype was only seen as a minority mutation in mixed infections, similar to previous findings (Osoti et al. 2022). An increase in the wild-type S436A/H SNP was observed compared to the previous study in 2019 by Osoti et al. (2022). Similarly, the *dhfr* I164L mutation increased as a mixed genotype since 2019, while *dhps* A581G showed similar frequencies with sampling done in 2019. The *Pfdhfr* 164 L mutation has been reported in Kilifi before (Kiara et al. 2009) and it showed high levels of resistance to pyrimethamine in vitro. There was also an increase in the prevalence of the lumefantrine-reduced susceptibility mutation *Pfmdr1* 184 F (Mungthin et al. 2010), from 83.7% (Osoti et al. 2022) to the current 98.5% mainly observed in mixed-genotype infections in the population.

Our study has several limitations, first, the CT cut-off of < 38.5% and the varying sample sizes across counties may have reduced the number of variants identified. Additionally, the application of a less than 5% cut-off during data analysis likely filtered out low-frequency variants, which could have affected the detection of rare mutations. Known controls for mixed k13 mutant and wildtype genotypes support the calling of low frequency mutations based on the limit of detection per sequencing run and should be included in sequencing runs to increase the accuracy in calling wildtype, mutant and mixed infection genotypes. Furthermore, mixed infections maintain parasite genetic diversity in asymptomatic infections and would be classified as a mutant phenotype, for instance for the *dhfr* and *dhps* genotypes. However, they potentially identify emerging resistant mutations in the population.

## Conclusion

Given the identification of 3 WHO validated artemisinin resistant mutations, continuous molecular surveillance of *P. falciparum* k13 is necessary. School children provide an effective sampling frame to act as an early warning system as they represent a broad and diverse cross-section of the community. Additionally, school children often have varied and extensive exposure to the malaria parasite. The Sulfadoxine-Pyrimethamine drug resistance marker mutations (triple *dhfr* and double *dhps*) are at a very high prevalence. Furthermore, the high prevalence of the *Pfmdr1* NF genotype, a lumefantrine resistance marker of the commonly used artemisinin combination therapy in Kenya warrants more investigations as to the continued effectiveness of lumefantrine. The emergence of C469Y, P533L and A675V mutations in the Kenyan school children population, highlights a potential risk of drug resistance emerging to the current first-line treatment of Artemether-Lumefantrine. Therefore, increased surveillance is essential, both in school children and in health facilities, to determine the prevalence of these mutations in clinical patients and their impact on treatment efficacy. TES studies are highly recommended in these regions where the validated mutations were identified to determine if the mutations are associated with clinical resistance. We note that many of the resistance mutations were present at low frequency and would not have been detected except with deep sequencing, as they were only present as minor alleles in mixed infections.

## Electronic Supplementary Material

Below is the link to the electronic supplementary material.

Supplementary Material 1

## Data Availability

Enquiries about using the data can be made to the KEMRI-Wellcome Trust Research Programme Data Governance Committee (dgc@kemri-wellcome.org). School based mRDT data enquires directed to RWS and the SNP data to ILO. The nucleotide sequence data reported in this paper are available in the GenBank database under the accession numbers: dhfr (PQ283609 -PQ283620), dhps (PQ283621-PQ283631) k13 469 (PQ283632-PQ283660) and mdr1 (PQ283661-PQ283673)Data are available under the terms of the Creative Commons Attribution 4.0 International license (CC-BY 4.0).

## Declarations

### Underlying data

This project contains the following data:

• Kenyansa, Victor, 2024, “FASTQ Files for the dhps and dhfr genes”, 10.7910/DVN/TRUDPB, Harvard Dataverse, V1.

• Kenyansa, Victor, 2024, “FASTQ Files for the k13-675 fragment gene”, 10.7910/DVN/Q7PD5P, Harvard Dataverse, V1.

• Kenyansa, Victor, 2024, “FASTQ files for the mdr1, ama1 and k13-469 fragment genes”, 10.7910/DVN/2TJH9F, Harvard Dataverse, V1.

• Kenyansa, Victor, 2024, “FASTA files for the k13-675 fragment gene”, 10.7910/DVN/O82JXI, Harvard Dataverse, V1.
