# Supplementary material for: Detection of low frequency artemisinin resistance mutations, C469Y, P553L and A675V, and fixed antifolate resistance mutations in asymptomatic primary school children in Kenya
Source: BMC Infect Dis. 2025 Jan 16;25:73. doi: 10.1186/s12879-025-10462-z (PMC11740484; doi:10.1186/s12879-025-10462-z)
Supplement: Supplementary file 1 — Supplementary Material 1 [file 12879_2025_10462_MOESM1_ESM.docx]

**Supplementary Table S1. Weighted frequency of resistance haplotypes for each gene and of infections containing mixed haplotypes**

| **Gene**  **(Codon)** | **Haplotype** | **n** | **Frequency (%)** |
| --- | --- | --- | --- |
| *dhfr* | IRNI | 600 | 91.06 |
|  | ICNI | 28 | 4.36 |
| (51,59,108,164) | IRNL | 21 | 3.31 |
|  | ICNL | 9 | 1.49 |
|  | NCSI | 1 | 0.15 |
|  | NRNI | 1 | 0.07 |
| *dhps* | SGEA | 638 | 84.36 |
|  | HGEA | 94 | 12.36 |
| (436,437,540,581) | SAKA | 15 | 1.86 |
|  | SGEG | 5 | 0.56 |
|  | SAEA | 2 | 0.34 |
|  | AAKA | 1 | 0.05 |
|  | AAEA | 1 | 0.02 |
| *mdr1* | NYT | 251 | 48.38 |
| (86,184,199) | NFT | 247 | 47.66 |
|  | NYS | 19 | 3.84 |
|  | NFS | 1 | 0.12 |
